# Global education movement: English as a second language teachers’ perceptions of integrating volatility, uncertainty, complexity, and ambiguity elements in lessons

**DOI:** 10.3389/fpsyg.2023.1007970

**Published:** 2023-03-22

**Authors:** Nur Syafiqah Yaccob, Melor Md Yunus, Dexter Sigan John

**Affiliations:** ^1^Sekolah Menengah Kebangsaan Dato’ Abdul Rahman Ya’kub, Melaka, Malaysia; ^2^Faculty of Education, Universiti Kebangsaan Malaysia, Bangi, Malaysia; ^3^Faculty of Language and Communication, Universiti Malaysia Sarawak, Kota Samarahan, Sarawak, Malaysia

**Keywords:** English as a second language lesson, ESL teaching and learning, global competence, global education, VUCA

## Abstract

Education evolves and progresses globally in a technology-driven world, highlighting the integration of VUCA elements of volatility, uncertainty, complexity, and ambiguity in lessons. Learners are molded to equip themselves with the world and real-life knowledge in their readiness to adapt to the VUCA world. Global education consists of preparing learners for the VUCA world. This study aimed to investigate the current English as a Second Language (ESL) teaching practices and the ESL teachers’ incorporation of VUCA elements in ESL lessons. This study was conducted quantitatively using a survey questionnaire. This study was administered to 30 ESL teachers from different secondary schools in a district in Malacca. The results from the questionnaire revealed that the ESL participants had positive perceptions toward the adaptation of VUCA in the current ESL lessons. Most of the ESL teachers agreed that they adapted VUCA elements into the activities during lessons, although some showed uncertainty about their knowledge and understanding of VUCA. From the high agreement levels in the findings, it can be concluded that the ESL teachers agreed that VUCA elements through problem-based, digital-based, collaborative, and challenging activities in English lessons are beneficial to assist students’ meaningful and autonomous learning. Based on these findings, implications were made for enhancing ESL teachers’ knowledge, understanding, and skills in adapting VUCA in lessons in response to global education demand.

## Introduction

Globalization plays a dominant role in shifting ESL lessons from a traditional approach to a more modern setting (Husin et al., 2016; Srinivas, 2019; Shliakhovchuk, 2021). Orazbayeva (2016, p. 2659) defines globalization as “an objective process and a form of modern society’s existence.” The onset of globalization in the 21st century has cultivated significant modifications and changes in educational endeavors (Saquing, 2018; Rafiq et al., 2019). Therefore, education systems worldwide have taken significant initiatives to adhere to the standard outlined by global education. According to Sinagatullin (2019), global education goals include

1. the preparation of individuals to live, work, and cooperate with others,
2. the development of students’ global competence, and
3. the development of individuals with creative and reflective personalities.

Generally, it affects the teaching and learning process in the new era, resulting in a beneficial situation for teachers and learners (Allen and White, 2017). Simultaneously, as an irreplaceable factor in educational sectors (Sert, 2015), teachers play a more vital role than teaching, facilitating, and monitoring (Wang and Du, 2016; Dobber et al., 2017; Rowan et al., 2019). Furthermore, teachers have a huge role in global education (Orazbayeva, 2016). It includes the shift from teacher-centered to learner-centered learning which is significant in strengthening students’ autonomy (Singh and Yunus, 2021). Canzittu (2022) agrees that they prepare learners for the real-working world by adapting to the actual environment in lessons.

In today’s so-called global age, English represents a medium of interconnectivity. Proficiency in the language profoundly influences individuals and communities worldwide (Wong and Yunus, 2021; Lin, 2020). English is an instructional language and international medium of communication in many countries where English is not the mother tongue (Sinagatullin, 2019). That is why learning English is significant among non-native English speakers. Mastery of the English language helps individuals compete with native English speakers and other foreigners (Yunus and Arshad, 2015; SiRicord and Shah, 2017; Seow et al., 2019). In today’s global education sphere, the English language is learnt through meaningful approaches such as problem-based learning, project-based learning, and the integration of authentic contexts to enhance learners’ English acquisition.

According to Watkins (2019), English lessons need to utilize meaningful learning styles that create social learning opportunities, equip learners to communicate, collaborate with others, and participate actively in challenging and competitive surroundings. English language learning must be consistent with the goal of global education, which is to produce holistic individuals and future generations. Similarly, in Malaysia, the new Malaysian Education Development Plan oversees an aspiration to equip students with adequate skills to create holistic individuals who are balanced, resilient, inquisitive, principled, informed, caring, patriotic, to become effective thinkers, communicators, and team players (Muhamad and Seng, 2019). Nevertheless, the education goal to produce a holistic future generation can be achieved when students learn practical knowledge that will assist their potential undertakings and careers. Overall, it is associated with the aim of education to provide meaningful English learning for students to gain successful learning outcomes and to create versatile thinkers of the future (OECD, 2016; Mansilla and Wilson, 2020). Therefore, English teaching in Malaysia has evolved to teach and learn the target language in a more relevant context.

This study focuses on the teaching and learning English as a second language (ESL) in Malaysia as there has been a lack of discussion on the integration of VUCA in ESL lessons in Malaysia. VUCA is the acronym for volatility, uncertainty, complexity, and ambiguity. According to Hadar et al. (2020), OECD and UNESCO have emphasized the importance of bracing learners for the VUCA world. Volatility is defined as changeable, unpredictable and the deviation from the expected that best describes the current era (Bennett and Lemoine, 2014; Maier et al., 2016). Uncertainty means indefinite, showing no exact methods or solutions to something (Bennett and Lemoine, 2014; Maier et al., 2016). Maier et al. (2016, p. 155) also affirmed that “when dealing with an uncertain future, a different conceptual approach to thinking about uncertainty is needed.” Hence, ESL learners need to develop their critical thinking and expand their views on the use of language in the future (SiRicord and Shah, 2017; Baimanova et al., 2020). On the other hand, complexity in the real world arises when there is difficulty in identifying and quantifying it (Bennett and Lemoine, 2014; Maier et al., 2016; Stover and Seemiller, 2017). Ambiguity challenges learners’ skills to predict and be clear on issues that are vague and subject to interpretation (Reeves and Reeves, 2015; Asmolov, 2018). Subsequently, English education should prepare ESL learners to face all these elements in the present and future.

Developing English proficiency is crucial, as it has become the language to gain excellent knowledge of the world (Lin, 2020). Besides, there is a need for a nexus between education and the fourth industrial revolution (4IR) (Reeves and Reeves, 2015; Seow et al., 2019). Thus, the combination of the 21st century and volatility, uncertainty, complexity, and ambiguity (VUCA) environment is an ideal approach for ESL teaching and learning practices (Seow et al., 2019). For that, Stover and Seemiller (2017) and LeBlanc (2018) suggest the incorporation of VUCA because it can create a meaningful learning environment. Wang (2019, p. 52) expresses that “the educational success in the 21st century focuses on competencies that go beyond cognitive development, such as the ability to critically seek and synthesize information and to direct one’s learning.” Likewise, learners need to prepare for real-life challenges due to the unprecedented complex challenges and problems in the world today (Bennett and Lemoine, 2014; Maier et al., 2016; Rowan et al., 2019; Seow et al., 2019). Du and Chen (2018) support the opinion that today’s environment is marked by the VUCA trend. It will enable students to learn better, faster, and more than they are accustomed to when dealing with complex challenges in the workplace.

To ensure VUCA’s smooth implementation, ESL teachers must be proactive (Kilic, 2015; Wang and Du, 2016) and implement various activities that target learners’ competency and skills in coping with real-life problems (Clary, 2015). Learning a language is not solely focused on the linguistic skills but also the background of the language and learning content. In that regard, English teachers must create meaningful learning experiences with interesting global content for ESL students (Zulkefly and Razali, 2019). Since VUCA is a new concept, its incorporation in ESL lessons has not been done much. Canzittu (2020) and Stein (2021) both proposed VUCA to be incorporated in education world. Seow et al. (2019), on the other hand, studied the incorporation of VUCA in learning pedagogy to the university students. In the Malaysian context, there is a lack of studies or little is known whether ESL teachers integrate VUCA in their lessons. Therefore, this study examined their perceptions of their ESL teaching practices and the integration of VUCA in language lessons. The research questions of the study are:

1. What are the current ESL teachers’ teaching practices in association with VUCA elements?
2. What are the ESL teachers’ perceptions on integrating VUCA elements in ESL lessons?

## Literature review

### The role of VUCA in ESL lessons

Today, VUCA elements are integrated into 21st-century teaching and learning for learners to work collaboratively, use technological tools, and develop their higher-order thinking skills (HOTS) in lessons (Chai and Kong, 2017; Gunawardena et al., 2017; SiRicord and Shah, 2017; Kusumastuti et al., 2019). According to Wright and Zhu (2018), a complicated world undergoing rapid globalization requires complicated thinking students. Primarily, issues pertaining to globalization and global education are pointing toward learners who are preparing themselves to face the working world. They need to deal with such conflicts and competition in today’s volatile era (Canzittu, 2022). In education, globalization is a major trend that promotes democratic reforms of different countries, with English being set as an imperative means of communication in the current global educational space (Sinagatullin, 2019). Thus, education in the 21st century should prepare learners to live, work and survive in the uncertain and risky real-life environment (Husin et al., 2016; Beno and Funke, 2017; Asmolov, 2018; Rafiq et al., 2019). It demands learners to develop their knowledge, competencies, and skills (Watkins, 2019; Ansarian and Mohammadi, 2018) rather than only focusing on academic performance. Thus, a significant and effective measure is integrating VUCA elements through technology-based activities (Reeves and Reeves, 2015), authentic problem-solving and project-based activities organized by teachers (Beno and Funke, 2017; Chai and Kong, 2017). These activities stimulate ESL learners to learn in a target context (Clary, 2015).

Gunawardena et al. (2017) describe the focus of second language teaching as developing essential language competencies to help learners communicate in another language. In Malaysia, English is learnt as a second language (ESL) in schools and educational institutions. Sukri et al. (2017) raise the issue of the rising rate of unemployment among university graduates, and it was found that graduates are not well-versed in English, despite obtaining excellent grade points. This implies that the students are not fully equipped to interact with their co-workers of diverse backgrounds at work due to their limited English proficiency and competency. Therefore, the concept of adapting VUCA in ESL lessons is significant as it provides a real-life platform for learners to use their target language where the issues are closely related to daily activities (Bennett and Lemoine, 2014; Seow et al., 2019). It is crucial for ESL teachers to integrate the elements of volatility, uncertainty, complexity, and ambiguity in their ESL activities. English is an important language for various purposes and learning the target language is to use it effectively and successfully. Lacking the ability to communicate messages and ideas effectively may cause misunderstandings (Watkins, 2019) and inefficiency at work in a more complex environment (Clary, 2015). The cross-cultural collaboration and interaction that occur require individuals to be more aware to their surroundings (Shliakhovchuk, 2021). Hence, Malaysia prepares her learners to have good English competency enabling them to work in a diverse environment and to participate as part of the world community. The adaptation of meaningful and skill-based learning is relevant in today’s classroom to achieve high-quality education (Chai and Kong, 2017). As a result, ESL learners who are actively involved in the learning process can use the language meaningfully and think critically. These are the crucial aspects of learning in the VUCA environment.

### The role of ESL teachers in the integration of VUCA elements

The integration of VUCA elements should begin at the school level to ensure learners’ readiness to immerse themselves in the VUCA environment. This is concurrent with global education, which aims to prepare students to live, cooperate, and collaborate with people from diverse backgrounds of a larger community (Sinagatullin, 2019). According to Seow et al. (2019), universities need to produce students who can adapt to the VUCA work environment due to the constantly changing work environment. Thus, the teachers must lead the lessons with approaches that benefit the learners (Clary, 2015; Kilic, 2015) and prioritize autonomous learning to enhance ESL learners’ engagement (Akbari, 2015; Srinivas, 2019; Watkins, 2019). It is imperative for the teacher to adequately understand how VUCA should be embedded in ESL lessons. By assessing their perceptions of VUCA elements and its integration into ESL lessons, the authorities and curriculum developers can further address this concept and develop an appropriate program for teachers to develop their understanding and skills of VUCA.

LeBlanc (2018) and Yunus and Arshad (2015) state that one of the language teachers’ roles is to create a supportive autonomous learning environment in the classroom, leading to learners’ meaningful learning in ESL lessons. Hence, the teachers’ role is of paramount in providing access to the VUCA environment in lessons that can help stimulate learners’ awareness to learn through problems (Clary, 2015; Maier et al., 2016; Dobber et al., 2017; Seow et al., 2019). You can design language instruction that provides both guidance and freedom for learners to understand the material and acquire critical thinking skills as they face more challenging and uncertain situations in the future. This concept is associated with the VUCA environment. Other than language competency, it is revealed that social–emotional competencies comprising self-awareness and regulation, well-being, social-awareness, interaction skills, empathy, and responsible decision-making are entailed in learners’ holistic development (Hadar et al., 2020). This strengthens the case for integrating VUCA elements into activities for learners and teaching in general.

OECD has envisioned for future generations to develop skills and attitudes that will assist them to succeed in a VUCA world (Hadar et al., 2020). Hadar et al. (2020) add that teachers must possess similar skills to navigate the ever-changing education landscape and cope with the challenges of today’s VUCA world. We are living in unprecedented times and teachers should possess sufficient pedagogical knowledge, skills, and dispositions of VUCA environment in education. Their understanding of future needs will be reflected in their readiness to support planning and the adaptation of the VUCA environment in education, although it seems challenging (Frank et al., 2014; Clary, 2015; Maier et al., 2016; Asmolov, 2018). The unpredictable changes in education, economy, business, politics, and various other sectors demand that individuals be prepared. It resonates with the educational reformation in most countries including Malaysia which has employed the Common European Framework of Reference or CEFR-aligned curriculum in the English language curriculum, broadening its trajectory to a more global standard. The CEFR-aligned curriculum introduces more international and global topics for language learners to learn the target language more effectively in authentic and real-life contexts (Chong and Yamat, 2021). Therefore, it is necessary for English teachers to have a strong foundation in subject matter, general pedagogy, pedagogical content, learners and learning, curriculum, and educational philosophies.

Malaysian ESL teachers must be confident and well-prepared to design the new VUCA environment to meet the current educational trends. Besides, it reflects the country’s education and management (Chai and Kong, 2017; Du and Chen, 2018). Additionally, they should constantly attend professional development courses to meet the 21st-century teaching demand (Clary, 2015; Kilic, 2015; Allen and White, 2017; Chai and Kong, 2017; Lo, 2019). Past studies by Rowan et al. (2019) and Lo (2019) suggest a revamp in the teacher education programs so it will develop their knowledge, skills, and attitudes to meet both local and global needs. However, for in-service ESL teachers, it is more convenient for the Ministry of Education (MoE) to create a well-structured program that specifically aims to develop the ESL practitioners’ VUCA understanding and skills in integrating VUCA in lessons.

## Methodology

### Participants

The study was conducted using a quantitative approach, using a survey questionnaire to collect data in accordance with the purpose of the study. A purposive sampling approach was employed to get better insights on the topics from the ESL respondents, who were the practitioners. The study results were not meant to be generalized to a larger population. This is due to the small sample size (Creswell and Creswell, 2018). The participants were 30 (female = 26, male = 4) in-service secondary school ESL teachers in a specific district in Malacca; Jasin. The population of ESL teachers teaching in the secondary school in Jasin was small, hence the small sample size. The respondents were from the same district but taught in three different types of school, namely Government Secondary School, Religious Government Secondary School and Religious-Government Assisted Secondary School. Their age range was between 21 and 50 years old, with a year and more experience in ESL teaching. The selection of these in-service teachers is to ensure that they have some ESL teaching experiences to enable them to respond to the survey items on teaching practices.

### Instrument

The questionnaire was designed according to the study’s aims to investigate ESL teachers’ perceptions on their teaching practices in relation to VUCA adaptation and their perceptions on the importance of integrating VUCA elements in ESL lessons. The questionnaire was developed through an online platform; Google Form, since the MoE has provided a Google account for all Malaysian teachers. The instrument consisted of 36 items, with 30 items on a 5-point Likert scale agreement. The ESL teachers were required to identify their perceptions on the topic using the Likert scale, 1 for Strongly Disagree, 2 for Disagree, 3 for Uncertain, 4 for Agree, and 5 for Strongly Agree. These items were divided into three sections: demographic information, the perceptions on current ESL teaching and lessons, and the perceptions on the importance of integrating VUCA in ESL lessons.

Prior to the distribution of the study, the questionnaire went through content and face validity checking by an expert in the area of study and the researcher’s supervisor. It was then pilot-tested on four teachers. The ESL teachers involved in the pilot study were not involved in the actual study. The purpose of the pilot test is to provide an initial evaluation of the items’ internal consistency, assess the duration of answering the questionnaire, and identify potential concerns (Creswell and Creswell, 2018). After obtaining the responses, the instrument was revised and edited accordingly.

### Data collection procedure

The actual participants were contacted *via* social networking sites from the district ESL teachers’ group on Telegram. An explanation of the survey study was provided together with a Google Form link to the questionnaire. It was vital to provide the respondents with sufficient information on the area of interest and on VUCA. Considering the ESL secondary school teachers’ heavy workload and other professional commitments, the respondents were given 2 weeks to complete the questionnaire. Their participation was not made compulsory as the study required willing respondents who would like to contribute to the findings by sharing their views quantitatively. The survey questionnaire using online Google Form was employed as it was easy for the ESL teachers to access.

The accumulated data were analyzed using Statistical Package for Social Sciences (SPSS 22.0) through descriptive analysis of mean score and standard deviation. The overall mean score was interpreted in four categories of the agreement level; low, medium-low, medium-high, and high (Alias, 1997 in Raduan et al., 2016) as shown in Table 1.

**Table 1:** Interpretation of the mean score.

Level	Mean score
Low	1.00–1.75
Medium low	1.76–2.50
Medium high	2.51–3.25
High	3.26–4.50

Source: Alias Baba, 1997 in Raduan et al. (2016).

### Findings

This section presents result on ESL teachers’ teaching practices in association with VUCA elements and their perceptions on integrating VUCA elements in ESL lessons. From this section onwards, ESL teachers will be referred to teachers only (Table 2).

**Table 2:** Teachers use of VUCA elements in ESL lessons.

Items	Mean	Std. deviation
I encourage students to relate the learned items with real-life situation	4.47	0.51
I make sure that students experience meaningful learning	4.37	0.49
I give students problems to solve	4.27	0.58
I apply collaborative learning in lessons	4.23	0.57
Students learn to think critically from the process of solving the problems	4.17	0.65
I incorporate authentic materials for activities in lessons	4.13	0.68
I am comfortable with my teaching and learning style	4.13	0.63
Students learn critical thinking skills from complex problems and activities in lessons	4.13	0.51
My students are comfortable with my teaching and learning style	4.10	0.71
Students learn to cooperate when completing the project	4.10	0.66
I allow students to use technology in lessons and for out-of-class activities	4.07	0.69
Students find the English lessons to be enjoyable	4.03	0.72
I give students project to complete	4.03	0.72
Students show better understanding when I use technology	3.93	0.78
I use technology in lessons and for out-of-class activity	3.77	0.73

### VUCA elements in ESL lessons

Based on the mean scores above, the teachers generally adopted VUCA elements in their teaching practices. First, the teachers included “real-life situation” (*M* = 4.47), “authentic materials” (*M* = 4.13) and “project” (*M* = 4.03) in the English language tasks. The results show that to introduce VUCA elements in ESL lessons, most of the teachers in this study carried out classroom projects based on the real-life situations that required students to use authentic materials. Such projects give students exposure to challenging real-life situations and their ways to respond to them. The teachers also added the elements such as “meaningful learning” (*M* = 4.37), teacher’s comfortability in teaching and learning style (*M* = 4.13), student’s comfortability in teaching and learning style (M = 4.10), and “enjoyable lessons” (*M* = 4.03) in their teaching approaches. These are indirect evidences that the teachers attempt to vary their teaching approaches that train students to adapt to the VUCA environment.

Students also find classes enjoyable and relevant as the teachers incorporated “problems” (*M* = 4.27), “process of solving the problem” (*M* = 4.17), and “critical thinking skills” elements (*M* = 4.13) in students’ learning. These results confirm that the teachers are aware of the VUCA elements where they employed problem-based learning in their ESL lessons. They viewed that this integration could enhance students’ critical thinking and problem-solving skills. Apart from that, the teachers also promoted collaborative learning in ESL lessons (*M* = 4.23) and projects (*M* = 4.10). In the 21st-century teaching and learning, using technology in VUCA environment is necessary. In that regard, both teachers (*M* = 3.77) and students (*M* = 4.07) used “technology in lessons” (*M* = 4.07). The teachers perceived that their students showed “better understanding” when they used technology in the ESL lessons (*M* = 3.93).

### English as a second language teachers’ perceptions on integrating VUCA elements in ESL lessons

The data from this section represent varied responses from ESL teachers’ perceptions regarding VUCA based on their knowledge, attitudes, and practices. Table 3 demonstrates the perceptions of integrating VUCA in ESL lessons.

**Table 3:** English as a second language (ESL) teachers’ perceptions on the knowledge, attitudes, and practices of VUCA elements.

Items	Mean	Std. deviation
Knowledge		
I know that applying VUCA will help students to deal with uncertain situations better	3.63	0.76
I know that 21st century learning is part of VUCA environment	3.60	0.93
I know how to apply VUCA elements in lesson planning and activities	3.40	0.97
I know that V in VUCA stands for volatile, U in VUCA stands for uncertain, C for complex and A for ambiguous	3.30	1.15
The MOE should provide more information on applying VUCA environment in lessons	4.33	0.61
Attitudes		
I believe that applying VUCA will improve students’ ability to solve complex and ambiguous problems	3.83	0.75
I have positive views regarding the adaptation of VUCA in ESL lessons	3.80	0.92
I believe that applying VUCA will help students to prepare for the real-working and volatile world	3.77	0.86
I do not think the VUCA elements suit my lessons and activities	2.30	1.09
Practices		
I do not see the needs to apply the VUCA elements in ESL lessons	2.17	1.12
I do not vary the activities in ESL lessons to suit the current needs for students and employers	2.17	0.99
I do not think that VUCA elements/environment should be integrated in ESL lessons	2.17	0.99
I do not create uncertainty and ambiguity to develop students’ higher order thinking skills (HOTS)	2.03	0.93
I do not give problems for my students to solve in ESL lessons	1.93	1.08

The results show that the teachers had positive perceptions of the knowledge, attitudes, and practices of VUCA elements. In terms of knowledge on VUCA, the teachers mostly agreed that the Ministry of Education Malaysia should provide sufficient information on the integration of VUCA in lessons (*M* = 4.33). This response explains the teachers’ willingness to explore VUCA and the information from the ministry would assist them in its implementation. The teachers also agreed that VUCA elements are to assist learners dealing with uncertain situations (*M* = 3.63) and a part of the 21st-century learning (*M* = 3.60). This agreement shows that the teachers want VUCA to be part of students’ learning due to its ability in preparing students for the unexpected challenges. The teachers also understood what VUCA stands for (*M* = 3.30) and this is an indication that the teachers are familiar with its concept.

Furthermore, the teachers also have optimistic attitudes on the integration of VUCA elements in their teaching approaches. The teachers applied VUCA to enhance students’ problem-solving skills (*M* = 3.83), positively viewed VUCA’s integration in ESL lessons (*M* = 3.80), and implemented VUCA to prepare students for the real-working and volatile world (*M* = 3.77). These results show that most of the teachers prepared students with challenging and practical activities in the ESL classrooms that aimed to strengthen their problem-solving skills. However, the teachers showed a moderately positive response to the negative statement, “I do not think the VUCA elements suit my lessons and activities” (*M* = 2.30). This result indicates that the teachers carefully selected the ESL activities to ensure they suit students’ learning needs.

For teachers’ perceptions of the practices of VUCA elements, all items in the questionnaire were phrased using negative statements that began with ‘I do not.’ Even so, the teachers perceived they needed to “apply VUCA elements,” vary “activities in ESL lessons,” integrate “VUCA elements/environment” in lessons, and pose “problems for my students to solve.” These responses confirm that most teachers carried various problem-solving activities in line with VUCA elements in their ESL lessons. A variety of challenging activities would develop students’ higher-order thinking skills.

## Discussion

The overall results reveal that most teachers adopted VUCA elements in their teaching practices and positively perceived its integration into their lessons. For the teaching practices aspect, the teachers incorporated VUCA elements in their ESL lessons. These lessons were conducted with various approaches such as authentic use of materials, problem-based and project-based tasks, HOTS-related activities and collaborative learning. From the results, most of the teachers preferred to apply “real-life situation,” “meaningful learning,” “problems” and “collaborative learning” in their ESL classrooms. From these findings, it can be derived that the teachers provided their students with authentic and relevant ESL activities to bridge the gap between the classroom and the real world. Yew and Goh (2016) explain that using problem-based learning as a pedagogical approach allows learners to learn while actively engaging with meaningful problems. This serves as a challenge for learners to apply learned knowledge and skills in dealing with real-world problems (Yew and Goh, 2016; Husin et al., 2016; Seow et al., 2019). Therefore, integrating problem-solving learning is seen as a window to bring learners closer to the unsolved real-world problems, issues, or challenges within organizations that trigger a sense of excitement, urgency, and importance in learning (Yew and Goh, 2016; Wright and Zhu, 2018).

Furthermore, the teachers were also aware of the role of collaborative learning in the ESL classrooms. Husin et al. (2016), Evans-Whipp et al. (2017), and Kusumastuti et al. (2019) state that the learners develop collaborative and critical thinking skills when they work with others to complete the authentic tasks. Collaborative interaction will further nurture learners’ second language acquisition, formally and informally (Gunawardena et al., 2017; Wong and Yunus, 2021). A similar approach is also useful in creating a better understanding among learners and enhancing their ability to cope with complex situations such as in VUCA environment. From these evidences, it is apparent that the teachers’ teaching practices are in line with the basic principles of global education that are holistic, humanistic, integrative, problem-solving, critical, and scientific approaches (Sinagatullin, 2019).

The teachers also had positive views on integrating VUCA elements in the ESL lessons. Their views are divided into three aspects: knowledge, attitudes, and practices. In terms of knowledge of VUCA, most of the teachers perceived that the Ministry of Education Malaysia should provide sufficient information. This revelation explains that the teachers needed more guidelines to properly implement VUCA in their lessons. Since the teachers were from three different types of schools namely Government Secondary School, Religious Government Secondary School, and the Religious-Government Assisted Secondary School, the focus on language learning might be different. Therefore, the Ministry of Education Malaysia should set a clear blueprint for the integration of VUCA elements in ESL lessons although the Malaysian secondary schools use the same Standard-Based English Language Curriculum. Lack of information and knowledge about how to implement VUCA results in teachers not having sufficient skills to effectively integrate VUCA in the classroom (Canzittu, 2022). Besides, the findings also showed that the teachers mostly applied the VUCA elements in their lessons to help their students deal with uncertain situation better. Due to the rapid development of globalization, it is essential for the education system to change and adapt to the new environment. In this context, teachers need to expose students to different topics, tasks, situations, and elements in ESL classrooms. As part of the shift to a global education system, the Malaysian Ministry of Education is responsible for reviewing the quality of in-service training programs in the areas that contribute to the development of excellent ESL teachers (Sukri et al., 2017).

The ESL teachers have shown positive attitudes toward VUCA as most of them perceived that integrating VUCA will improve the students’ problem-solving abilities. This is significant in today’s 21st-century learning industry because the higher-order thinking skills approach is critical in Malaysian settings. HOTS has been increasingly highlighted in the Malaysian education system, with the introduction of a new education policy in 2013 which emphasizes the development of HOTS, as opposed to memorization of facts and information (Chua, 2015; Ministry of Education Malaysia, 2020). It trains learners to be proactive in their learning as teachers guide student learning through problem analysis, exploratory pathways, and collaborative sessions with peers (Wang and Du, 2016; Yew and Goh, 2016; van Leeuwen and Janssen, 2019). From the results, ESL teachers’ optimistic perceptions can be seen in their agreement in incorporating VUCA strategies into the lesson plan. It can be concluded that most ESL teachers agreed that they have created a classroom environment that promotes flexibility and critical thinking through multiple activities, problems, and VUCA-driven learning. Through activities and meaningful practices, ESL teachers can help students better cope with uncertainty and ambiguity, which is essential in a rapidly changing world (Choudhury, 2017). While the 4IR is heavily focused on digitalization, technological advancement and integration, Industry 5.0 will increase collaboration between humans and intelligent systems where humans will take the creative sides responsibilities to elevate quality (Paschek et al., 2019). Overall, Malaysian ESL teachers’ VUCA practices in the classroom are consistent with the Global Learning goal of producing holistic, competitive, responsible, and critical individuals making decisions. More conscious emphasize of the VUCA integration is needed to enhance learners’ capabilities to work in the future industry.

## Conclusion

This study on teachers’ perceptions toward the integration of VUCA in ESL teaching and learning showed that teachers have positive perceptions toward VUCA implementation. In order to create a meaningful learning environment, teachers must design ESL lessons that are real-life and problem-based. Systematic guidelines from the Ministry of Education Malaysia are needed to ensure the effective implementation of VUCA elements in ESL lessons. This study is significant because it has added to the literatures on how to incorporate VUCA elements in ESL lessons, especially in secondary school contexts. Besides, it can serve as a clear guideline for the education officers, policymakers, and stakeholders to design a framework to assist teachers in applying VUCA in their classrooms. However, it should be noted that this study is limited to teachers in a small district in Malacca, Malaysia. The usage of VUCA in other school parts in Malaysia has not been investigated and should be the focus of future research.

## Data availability statement

The original contributions presented in the study are included in the article/supplementary material, further inquiries can be directed to the corresponding author.

## Ethics statement

Ethical review and approval was not required for the study on human participants in accordance with the local legislation and institutional requirements. Written informed consent from the [patients/participants OR patients/participants legal guardian/next of kin] was not required to participate in this study in accordance with the national legislation and the institutional requirements.

## Author contributions

All authors listed have made a substantial, direct, and intellectual contribution to the work and approved it for publication.

## Funding

Funds received for open publication fees, from my institution (grants), National University of Malaysia/ Universiti Kebangsaan Malaysia with grant no. GG-2022-031.

## Conflict of interest

The authors declare that the research was conducted in the absence of any commercial or financial relationships that could be construed as a potential conflict of interest.

## Publisher’s note

All claims expressed in this article are solely those of the authors and do not necessarily represent those of their affiliated organizations, or those of the publisher, the editors and the reviewers. Any product that may be evaluated in this article, or claim that may be made by its manufacturer, is not guaranteed or endorsed by the publisher.
